# Nanomaterial-Based CO_2_ Sensors

**DOI:** 10.3390/nano10112251

**Published:** 2020-11-13

**Authors:** Marwan Y. Rezk, Jyotsna Sharma, Manas Ranjan Gartia

**Affiliations:** 1 Department of Petroleum Engineering, Louisiana State University, Baton Rouge, LA 70803, USA; mrezk2@lsu.edu; 2 Department of Mechanical and Industrial Engineering, Louisiana State University, Baton Rouge, LA 70803, USA; mgartia@lsu.edu

**Keywords:** nanomaterials, CO_2_ monitoring, gas sensing

## Abstract

The detection of carbon dioxide (CO_2_) is critical for environmental monitoring, chemical safety control, and many industrial applications. The manifold application fields as well as the huge range of CO_2_ concentration to be measured make CO_2_ sensing a challenging task. Thus, the ability to reliably and quantitatively detect carbon dioxide requires vastly improved materials and approaches that can work under different environmental conditions. Due to their unique favorable chemical, optical, physical, and electrical properties, nanomaterials are considered state-of-the-art sensing materials. This mini-review documents the advancement of nanomaterial-based CO_2_ sensors in the last two decades and discusses their strengths, weaknesses, and major applications. The use of nanomaterials for CO_2_ sensing offers several improvements in terms of selectivity, sensitivity, response time, and detection, demonstrating the advantage of using nanomaterials for developing high-performance CO_2_ sensors. Anticipated future trends in the area of nanomaterial-based CO_2_ sensors are also discussed in light of the existing limitations.

## 1. Introduction

Carbon dioxide (CO_2_) is vital to life on Earth. It is also pivotal for many biological and industrial processes. The concentration of CO_2_ in Earth’s atmosphere is currently close to 412 parts per million (ppm) which represents a 47% increase since the beginning of the Industrial Age, when the concentration was near 280 ppm, and an 11% increase since 2000, when it was near 370 ppm [1]. The rising concentration of CO_2_ in the atmosphere is driving up surface temperatures and causing ocean acidification, making it one of the primary climate change contributors [2]. Moreover, extended exposure to high CO_2_ concentration can also be lethal for human beings. Thus, sensing and monitoring of CO_2_ is fundamental to gaining knowledge about CO_2_-affected mechanisms and controlling them.

The conditions in which CO_2_ is monitored dictate the method and the materials that can be used in the sensor [3]. A variety of sensors have been developed based on different sensing principles including optical absorption [4,5,6], electrical resistance [7,8,9,10], field-effect transistors [11,12,13], and amperometry [14]. However, conventional CO_2_ sensors have several limitations such as higher cost, heavier weight, bigger size, and less durability [15]. To enable the widespread adoption of CO_2_ sensors in many aspects of modern society, inexpensive mass-producible devices are needed that offer simplicity, robustness, and ultralow power demand. Furthermore, there is a need for an accurate and reliable sensor that operates at harsh conditions of pressure and temperature such as in deep oil wells or nuclear reactors. To meet these needs, there has been a growing interest in recent years in nanomaterial-based CO_2_ sensors due to their cost efficiency, durability in harsh conditions, and stability [16,17]. The use of nanomaterials has been shown to improve traditional sensing techniques in terms of sensitivity, stability, response time, and selectivity [18]. The aforementioned benefits of nanomaterials are often ascribed to their high surface to volume ratio, high surface energy, quantum size effect, and high electron conductivity [19].

Nanomaterials used for sensing may consist of organic, inorganic or hybrid components. In CO_2_ sensing, it is desirable to use materials with functional groups that display application interaction. Organic materials exhibit many desirable properties such as mass transport, chemical reactivity, and gas diffusivity in the context of CO_2_ sensing. On the other hand, inorganic materials show mechanical stability, conductivity, and optical properties [20]. Hybrid materials have also been explored for CO_2_ sensing in which unique chemical conjugates of organic and inorganic components are brought together by specific interactions for synergistic improvement of their functional properties [21].

The main thrust of this mini-review is to document the most significant work done in the area of nanomaterials-based sensing of CO_2_, primarily in the free gas state, in the last two decades. On the basis of the key sensing mechanism, CO_2_ nanosensors can be categorized based on electrochemical principle (which includes chemiresistive, capacitive, and inductive sensors), and based on optical sensing (which includes surface plasmonic resonance, colorimetric, refractometric, and non-dispersive infrared sensors). These broad categories of sensors are discussed in the following sections in terms of their sensing mechanisms and the most significant nanomaterials used. Their common applications, inherent drawbacks, and key performance metrics are compared to serve as a guide for selecting the CO_2_ sensor appropriate for meeting the application-specific demands and requirements.

The manifold application fields as well as the huge range of CO_2_ concentrations to be measured make CO_2_ sensing a challenging task. In view of the great diversity of CO_2_ measurement tasks, this mini-review article is not intended to provide solutions for all possible measurement problems. Rather, its scope is restricted to surveying the current developments in the field of nanomaterial-based CO_2_ sensing, highlighting their strengths, weaknesses and the most representative application space. Anticipated future trends in the area of CO_2_ sensing are also discussed in light of the aforementioned comparison.

## 2. Electrochemical CO_2_ Sensors

**Figure 1:**
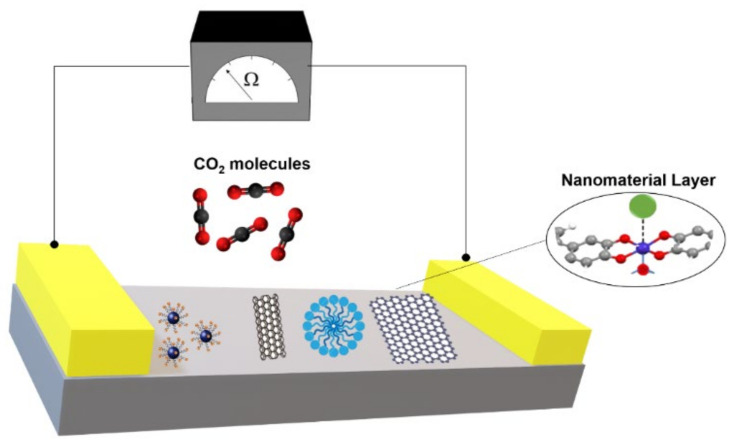
Sensing mechanism of electrochemical CO_2_ sensors via different nanomaterials.

The main working principle of these types of sensor is the variation in electrical properties upon chemical interaction with CO_2_. The most commonly used gas sensors in this category are chemiresistive which measure the change in resistance, and capacitive, which measure the change in capacitance corresponding to the change in the CO_2_ concentration [22]. The simplicity of this sensing technique as well as its cost-effectiveness were the main incentives in its wide development. The most widely used type of nanomaterials in this area are inorganic metal oxides (such as ZnO, SnO_2_, CuO, and CdO) where p-n-type can act as the base material for the sensing layer. When CO_2_ comes in contact with the semiconductor nanomaterial layer a surface interaction may occur through oxidation/reduction, electron charge transfer, adsorption, or chemical reaction [23]. The chemical interaction of the adsorbate (CO_2_) and adsorbent semiconducting nanomaterial causes a charge depletion layer with upward bending energy bands that lead to change in electrical properties [24,25]. This is represented in the schematic in Figure 1.

It has been reported that the sensitivity of such sensors mostly depends upon the nanostructure’s porosity [26,27,28]. At dry air conditions, the oxygen species chemisorbed on the surface of the sensing nanomaterial causes an electron depletion layer which creates a Schottky barrier [29,30,31]. The interaction between CO_2_ and chemisorbed oxygen species gas off carbon monoxide and electrons. Subsequently, this reaction decreases the Schottky surface barrier which increases conductivity. CO_2_, which is normally regarded as reducing gas except in some work it has been reported as oxidizing gas [32,33,34], increases the resistivity upon interaction with oxygen species [35]. Furthermore, it has been observed that high surface area and increased uniform pore size distribution make a great contribution to sensitivity as well as response time [36]. The sensor response (S.R) is measured as a function of change in resistance as shown by the equations below [37,38]:

(1) $$\mathrm{Reducing}\;\mathrm{gases}\;S.R\;=\;\frac{R_{a}-\;R_{g}}{R_{g}}\;\mathrm{Oxidizing}\;\mathrm{gases}\;S.R\;=\;\frac{R_{g}-\;R_{a}}{R_{a}}$$

where *R_g_* is the resistance of the sensitive nanomaterial layer in the presence of CO_2_ while *R_a_* is the resistance of the sensing layer in air. Studies have shown that the high surface to volume ratio of nanomaterials yields a quicker response time [39,40]. Similarly, the presence of an oxygen-rich background while sensing CO_2_ concentration increases the sensor’s response while the presence of amine functional groups can hasten the recovery time at varying temperatures [36,41,42]. CO_2_ as a strong Lewis acid strongly tends to accept additional pairs of electrons from primary and secondary amines that are strong Lewis bases. The aforementioned reaction is a covalent bond that occurs due to chemisorption of CO_2_ and the sensing nanomaterial. In assessing cross-sensitivity to identify the sensors’ specificity, some works have demonstrated the dependence of CO_2_ sensing on ambient humidity [43,44]. CO_2_ is known to hinder proton diffusion by blocking Grotthus pathways in the sensing nanomaterial layer [45,46]. These aforementioned phenomena cause less proton diffusion, and increased charge transfer resistance contributes to the overall sensitivity.

**Figure 2:**
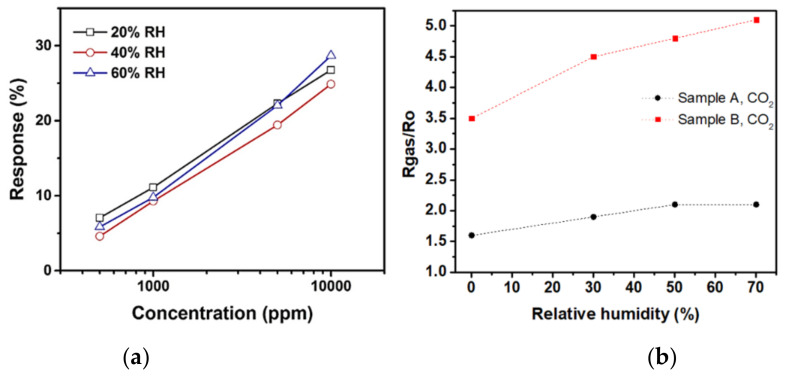
(**a**) Humidity inhibits sensor’s response (modified from [49]), (**b**) humidity enhances sensor’s response using LaOCl by two different synthesis techniques sample A (simple oxidation)–sample B (sol-gel) (modified from [48]).

An additional parameter that has enhanced CO_2_ sensitivity is the metal oxide doping. Based on the choice of material, if the sensing nanomaterial and the dopant can create a p–n junction, this will generally increase the resistance baseline. Furthermore, increasing doping from 0% to a specific threshold can diminish both response and recovery times. However, surpassing this threshold can have counter-effects in terms of sensitivity, response, and recovery times [7]. Some of the studies conducted CO_2_ sensing experiments in the presence of oxygen concentrations in the atmospheric background. However, in other published works [42,47] the sensitivity of nanomaterials to CO_2_ was not affected in the absence of oxygen. Similarly, the effect of humidity on the sensing nanomaterial was identified as debatable based on the nanomaterials’ selection and other factors. In some cases, humidity acted as a sensor response enhancer due to favorable CO_2_ adsorption [48]. In other cases, the inhibited sensor performance was attributed to the consumption of huge number of oxygen ions. This debatable humidity effect is illustrated in Figure 2 based on published studies.

Despite the varying temperatures at which CO_2_ sensing took place, no chemiresistive sensor was investigated beyond 450 °C in dry air [50]. The application of this type of sensor has been extended in different fields including food processing and agriculture industry [51], medical diagnosis [52], and environmental monitoring [53,54]. Table 1 summarizes the most recent and significant work undertaken in nanomaterial-based chemiresistive and capacitive CO_2_ sensors.

## 3. Optical Sensors

### 3.1. Surface Plasmon Resonance (SPR) Sensors

**Figure 3:**
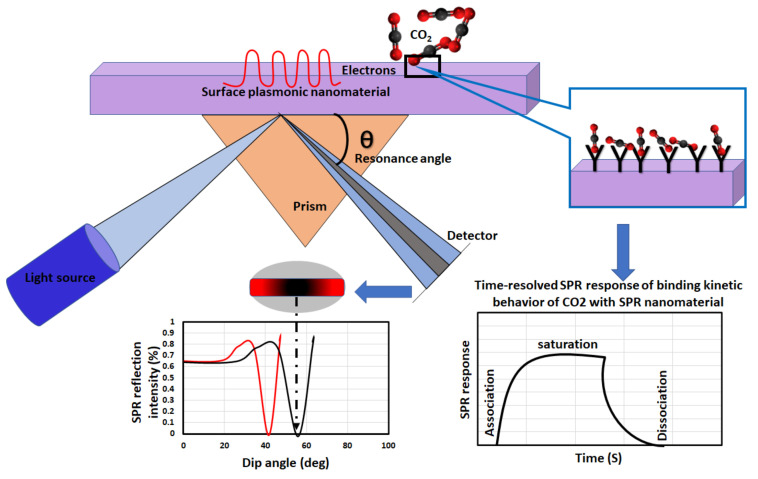
Mechanism of surface plasmon resonance (SPR) nanomaterial sensing for CO_2_ (modified from Patil et al., 2019 [69]).

The surface plasmon resonance (SPR) effect takes place when incident light, at a particular angle of incidence, causes resonant oscillation of electrons at the interface of two media [60]. The high sensitivity of the angle of incidence to the refractive index alterations is the basis of CO_2_ sensing in terms of adsorption and desorption. The changes in the effective refractive index of the sensing nanomaterial indicate a change in the concertation of CO_2_. In other words, these phenomena could be explained by what is referred to as the Kretschmann configuration. A few materials that exhibit negative real and small imaginary dielectric constant (Cu, Al, Au, and Ag) are the only materials showing surface plasmon capability [61]. The basis of this technique is the interaction of nanomaterial with the incident light. Owing to their superior optical properties, the absorption of incident light in nanomaterials can result in enhanced localized electric field at the localized surface plasmon resonance (LSPR) frequency. On metallic nanoparticles at the quantum size the conduction band is discretized which further enhances charge transfer [62]. In this detection scheme, a light source is used to illuminate a plasmonic nanomaterial. A portion of the light is reflected off the surface of the plasmonic nanomaterial [63,64] while a portion of light is absorbed by the surface electrons at a unique angle called the angle of resonance. This results in electronic resonance where they are called surface plasmons [65]. Surface plasmon resonance is a condition that alters the dielectric constant adjacent to the nanomaterial’s layer [66,67,68]. The sensitivity of surface plasmons results in a loss in the intensity of the reflected beam. The location and the shape of the observed dip in the SPR reflection intensity curve can be used to conclude information about the sensor’s surface. The kinetics of CO_2_ binding with the sensing material can also be studied through a time-resolved SPR response curve. In this curve, as CO_2_ interacts with the sensing material, the response increases as the binding of CO_2_ with the sensing material increases and once the system reaches equilibrium, CO_2_ starts unbinding or dissociating. The overall sensing mechanism is illustrated in the schematic in Figure 3.

The nanomaterials most used for plasmonic sensing of CO_2_ are carbon nanotubes (CNTs). The main reason for this is that the high selectivity of CNTs to CO_2_ at room temperature among other gases was ascribed to the high affinity of CNTs to CO_2_ causing high electron density and hole depletion [70,71,72]. However, the use of CNTs as plasmonic or optical sensors is still limited because they have an excitation regime in the infrared (IR) and ultraviolet (UV) regions and cross-sensitivity. In addition, some works have indicated that a need to improve reversibility and selectivity when sensing CO_2_ at room temperature [73]. Since this type of sensor usually shows non-toxicity with high sensitivity at low cost, it is widely used in biosensing application and pharmaceutical analysis [74,75].

### 3.2. Colorimetric Sensing

**Figure 4:**
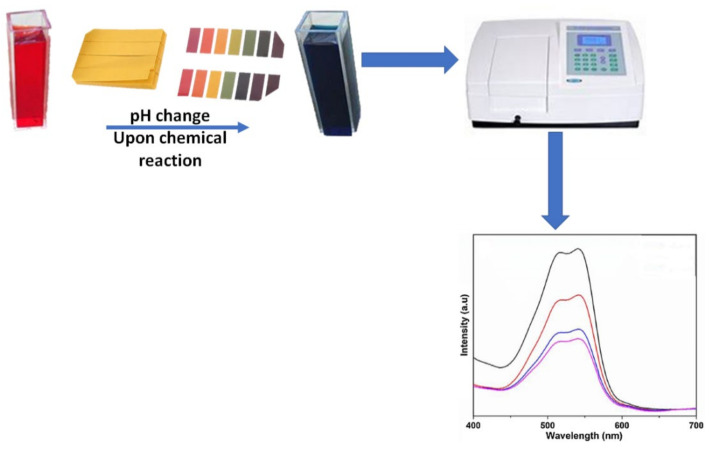
Schematic of colorimetric CO_2_ sensing. The pH change can be observed on the litmus paper or in the cuvette where the intensity is measured via UV-Vis spectroscopy.

Colorimetric CO_2_ sensing is a technique in which the sensing material (such as pH sensitive dyes or quantum dots) exhibits a color change upon chemical reaction or adsorption of CO_2_. Colorimetric sensors have been commonly used as semi-quantitative or qualitative non-invasive sensing technique in biological applications [76]. Their application is also reported for soil evaluation in carbon capture and storage sites [77]. The sensing technique is based on visual detection of the change in color due to a change of intensity at a specific wavelength on a dye. Depending on the material used, this sensing technique can be fully reversible or non-reversible [76,78]. In some cases, the Beer–Lambert law has been used to report differences in gas concentrations [79]. The aforementioned sensing technique is represented in Figure 4, where the pH change is depicted in response to CO_2_ with specific intensity.

The colorimetric nanomaterials sensing used to detect CO_2_ so far have been either complementary with fiber optic sensing or with dyes. Chu et al. [80] investigated the use of coarse silica nanoparticles (200 nm) as a phase transfer agent in fiber optic sensor. It was concluded that their proposed sensor had higher sensitivity and better linearity with slower response time compared to similar sensors. The enhanced sensitivity was ascribed to the high surface-to-volume ratio of silica nanoparticles.

**Figure 5:**
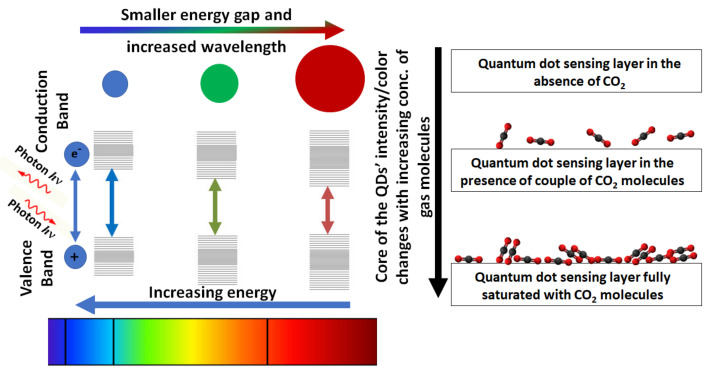
Quantum dots’ (QDs) response to CO_2_ sensing based on size, wavelength, and energy.

Quantum dots (QD) have been receiving much attention in gas sensing during the last decade. Specifically, in the area of colorimetric sensing, QDs are favorable in comparison to dye molecules owing to their quantum confinement as well as immunity to electromagnetic interference, narrow emission, wavelength size dependency, and superior photostability [81]. QDs are semiconductor nanocrystals (few nanometers in size) in which quantum confinement effects are proved. In other words, they are a group of nanocrystals in which electrons and electron-hole pairs are tightly confined. Quantum dots are either synthesized from semi-conductor compounds such as PbSe, CdSe, or PbS or they can be made simply of a single element such as germanium or silicon [82,83,84]. QDs are known for their unique optical and plasmonic properties due to their increased energy band gap as size decreases. Due to the energy quantization effects, quantum dots are capable of emitting colors more accurately. Upon reaction with CO_2_ and pH change in the presence of light, a photon hits an electron at the valence band. The excited electron moves from the valence band to the conduction band then relaxes again to a lower energy band by releasing a photon that matches the energy difference. This difference in energy corresponds to the color shift. The color shift would be affected by both the chemical reaction as well as the size of the quantum dot. Larger dots have smaller energy between bands with longer wavelengths and vice versa, as illustrated in Figure 5. The CO_2_ layer adsorbed on the surface of the QD will have an impact on the bandgap. However, the nature of this effect depends on the material of the QD and the type of interaction occurring with CO_2_. Since QDs have not been widely used in CO_2_ sensing it is hard to claim whether it would enhance the band gap or not. Many things could happen upon the adsorption of CO_2_ on the QDs’ surface. Firstly, the adsorption can cause a change in the intensity of the resonance. Secondly, Jin et al., 2006 [85] showed that the presence of a new layer of a different material on the surface of QD can cause a red shift due to a decrease in the bandgap. On the other hand, other researchers such as Joo et al., 2018 [86] proved that the adsorption of other chemical groups can cause surface defect passivation. This can result in a blue shift due to increasing bandgap. Furthermore, other researchers including Saha and Sarkar, 2014 [87] observed a bowing effect while increasing the concentration of the sorbed layer on the surface of QD. The effect of organic as well as inorganic passivation of QDs has been reviewed [88] with no sufficient referencing to the type of interaction of CO_2_ with QD.

### 3.3. Refractometric Fiber-Optic Sensors

**Figure 6:**
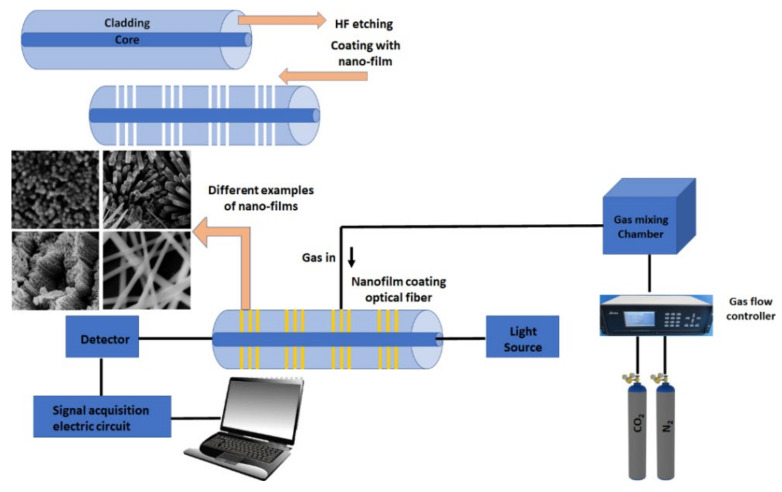
The use of nanomaterials thin film in fiber optics.

The use of fiber-optic CO_2_ sensing is quite prominent in environmental monitoring as well as aquaculture industry applications [89]. Fiber-optic sensing can be integrated with a variety of sensing platforms such as SPR sensing [90,91]. In SPR fiber optics, the core acts as the prism in the Kretschmann configuration. The same plasmon excitation mechanism is applied as in the prism-based setting. At a specific interval, the cladding is etched and the core is coated with the plasmonic nanomaterial [67]. A plethora of work has been done in CO_2_ interaction of nanomaterial coated over an unclad or clad etched fiber core causing a variation in the refractive index of the sensing material [92]. However, there are some drawbacks to SPR fiber optics including the integration of SPR into a multiplexed platform and challenges related to the limit of detection [93]. Shivananju et al. [94] developed a clad-etched fiber Bragg grating (FBG) with polyallylamine-amino-carbon nanotube coated on the surface of the core for detecting the concentrations of CO_2_ gas at room temperature over a wide range of concentrations (1000–4000 ppm). A reversible and reproducible linear response of Bragg wavelength shift was observed at a limit of detection (LOD) of 75 ppm. On the other hand, it was possible to make use of a metal-organic framework (MOFs) nano-porous structure in the CO_2_ optical-fiber sensor [92]. The main sensing material used was Cu-BTC (Cu-benzene-1,3,5-tricarboxylate) was coated over unclad core single-mode optical fiber. At the tested sensing region (5 cm long), this material recorded LOD of 20 ppm and a figure of merit (FOM) of 100 ppm. Reversibility investigation for the aforementioned material was not investigated [95]. In addition, other materials such as CNTs were coated on the surface of optical fibers and results were compared with an ordinary CNT uncoated optic fiber. The results proved enhanced selectivity and sensitivity due to the increased surface to volume ratio and CO_2_ reduced activation energy when interacting with CNTs [96]. A generalized simple schematic of optical fiber-based CO_2_ sensing using nanomaterials is represented in Figure 6.

Fiber optic-based gas sensors evaluates the concentration of gas as a function of the change in the refractive index (RI) [97]. Upon detection of CO_2_ concentration and changes in RI, frequency shifts of optical resonance are observed. Some of the drawbacks noted for these types of sensor are non-reliability, poor selectivity, and contamination issues. In recent work in this field, the aforementioned technique was merged with nanomaterials and used under the same umbrella. This work was motivated by the need for remote sensing, lessened noise interference, and a good candidate for CO_2_ sensing at harsh conditions [98]. Generally, the cladding of the fiber is etched using HF (hydrofluoric acid) to expose the core of the optical fiber. Then a sol or a thin film is coated in the etched place preferentially with porous nanostructures film. The increased surface to volume ratio as well as high porosity of nanomaterials help to increase the sensitivity.

### 3.4. Non-Dispersive Infrared Sensors

**Figure 7:**
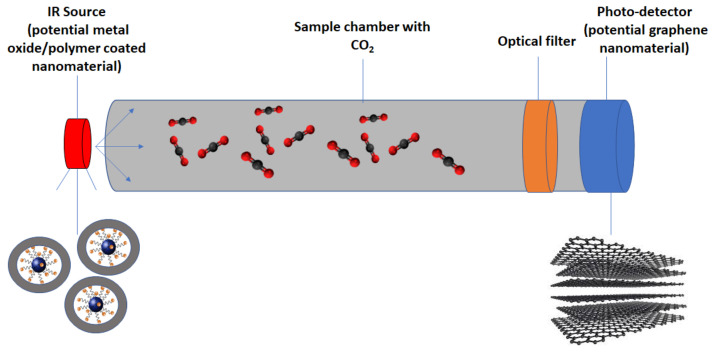
Schematic of non-dispersive infrared (NDIR) CO_2_ sensing with potential nanomaterial integration.

Optical detection based on the non-dispersive infrared (NDIR) method is a well-known and established concept for detecting gases. It is very common to use NDIR in monitoring the indoor air quality as well as automotive applications [99,100]. This sensing technique makes use of shining an infrared (IR) source on a sample in a chamber with an optical filter and an infrared detector, as shown in the schematic in Figure 7. Carbon dioxide can be easily identified by infrared spectroscopy because it is an infrared active molecule, which absorbs a 4.24 µm wavelength [15]. The light passes through the optical filter to obtain shorter wavelengths that are attenuated and longer wavelengths that are transmitted. If the CO_2_ is below the detection limit or absent, the detector will match the intensity to the reference level [100,101]. The IR intensity at the detector will reduce in accordance with an exponential relationship known as the Beer–Lambert Law: $I=I_{0}e^{kP}$, where *I* is the intensity at the detector subsequent to optical filtering, *I_0_* is the initial intensity prior to interacting with gas, *k* and *P* are the absorption coefficient and the gas concentration, respectively [102,103].

A few studies have reported the use of nanomaterials to improve either the IR emitting source [104] or the photodetectors [105]. Muller et al. [104] showed that IR emitter integrated with Pt-on-Si-needles demonstrated a 2.6 times higher IR emission without wavelength-dependent interference patterns as compared to an uncoated Si-based emitter at the same membrane temperature. Koppens et al. [105] evaluated state-of-the-art photodetectors based on graphene and other nanomaterials such as plasmonic nanoparticles. Similarly, Pusch et al. [106] investigated replacing the ordinary standard thermal emitter with CMOS nanoplasmonic tungsten crystal. They discovered that the sensitivity and signal to noise ratio to CO_2_ increased by 400%. This enhancement was attributed to slow-wave plasmonic lattice resonance in addition to elevated plasmon to light coupling.

Although this type of sensor has high accuracy, allows fast measurements and has a good long-term stability, even after decades of optimization, some inherent drawbacks of its optoelectronic mode of operation remain unresolved such as device complexity, power consumption, scalability, and cost [107,108]. Furthermore, the detection limit and sensitivity are highly dependent on the power-related parameters such as light intensity. A key issue that has not been well addressed in the literature is the efficacy of CO_2_ selectivity among the interference of adsorption bands [109]. Table 2 summarizes some of the recent significant work done in optical CO_2_ sensors using nanomaterials.

## 4. Challenges and Anticipated Future Trends

Owing to their unique properties, nanomaterials are emerging as key players in improving CO_2_ sensing technology. However, many challenges remain in catering to the diverse sensing demands. For example, CO_2_ selectivity remains problematic in the different types of CO_2_ sensor discussed here. The main concept through which CO_2_ selectivity is obtained nowadays is through the use of different gases’ working temperatures. In other words, at a specific range of temperature, one of the gases gives a higher response as compared to other gases due to chemical or physical interactions on the surface of the nanomaterial. This “lock and key” model is a widely used technique in biological fields where a sensing material has high specificity to lock on to the intended measurand. There is also a need for durable, low-maintenance, real-time sensors particularly in harsh environments, such as in a borehole and subsea. Thus, future work in nanomaterials should stack up that need. Multi-purpose sensing is also a fast-developing area, where the same sensing element is used for measuring multiple parameters. Some applications with high temperature requirements, such as in nuclear reactors would require sensors that can operate in range of 600–700 °C. More work in the future is required to enhance the temperature tolerance of these nanosensors. However, the selection of the elements in these composites seems non-systematic that is based on haphazard selection and trial and error. Therefore, there is a need for more basic studies and modelling to conceptualize the interaction of CO_2_ with different materials’ families.

Nanomaterial CO_2_ sensing is an interdisciplinary field that necessitates the identification of several parameters before deciding which nanosensor type to use. These parameters include but are not limited to dynamic sensing range, temperature, humidity, response, and reversibility. In addition, some other considerations such as size and cost should also be considered for successful CO_2_ sensing. A marketplace comparison of the commercially available CO_2_ sensors reveals useful insights [118]. Firstly, electrochemical sensors are typically less durable than optical sensors. In addition, more work should be done in this field when it comes to cross-sensitivity. While, chemiresistive CO_2_ sensors continue to be popular for a variety of applications due to their easy handling and greater tolerance to humidity and temperature changes. Secondly, the use of colorimetric and SPR is not as common as electrochemical ones due to the reduced accuracy in quantitative measurements. Thirdly, the use of refractometric CO_2_ sensing appears to be an emerging field with great potential since it can reach inaccessible areas and still measure with high accuracy. Multiplexing remains a topic that needs to be addressed in this field to make it more useful and achieve maximum performance for its cost. Finally, NDIR is a long-lasting type of sensor and it can measure CO_2_ at high concentrations with good selectivity. However, it can be affected by temperature and humidity. The authors think that nanomaterials have demonstrated tremendous potential in improving CO_2_ sensing towards higher efficiency and accuracy and although opportunities of improvement remain, nanomaterial-based sensing will continue to be a growing research priority.

## 5. Conclusions

This review summarizes the most significant work done in the area of nanomaterial-based CO_2_ sensing in the last two decades. The main sensing techniques were categorized by their sensing mechanism under two main categories and the role of nanomaterials in each sensing technique was highlighted. Their common applications, inherent drawbacks, and key performance metrics are compared to serve as a guide for selecting the CO_2_ sensor appropriate for meeting the application-specific demands and requirements.

## Figures and Tables

**Table 1:** Most significant recent work done in nanomaterial-based electrochemical CO_2_ sensors.

Nanomaterial Used	Dynamic Range (ppm)	Response/Recovery Time (s)	Measurand	Temperature (°C)	Remarks
CuO nanoparticles [55]	400–4000	720 @ 0% R.H 500 @ 20% R.H	Surface charge Surface species	25	R.H ↑ – CO_2_ diff ↓
Bi_2_O_3_ nanostructures [29]	10–100	132/82	Resistance	25	S.A/porosity ↑ –adsorption/desorption of CO_2_ ↑
Inorganic silica nanoparticles [15]	400–2000	>60/>150	Capacitance	30, 42, 58	Amine groups ↑–recovery time ↓
p-CuO/n-ZnO hetero-surfaces [56]	1000	76/265	Resistance	100–400 Optimum = 320	lattice mismatch ↑–electron transfer ↑
La_1−*x*_Sr*_x_*FeO_3_ (O9XS0.3) [47]	2000, 4000	660/300	Resistance	200–500 Optimum = 380	R.H ↑– sensitivity ↓
YPO_4_ nanobelts [57]	200–800	136/N/A	Impedance	350, 400	T > 400 °C–NS ↓
100 nm fumed silica [36]	500–3000	>120	Capacitance	38–65	R.H < 60%, T > 46 °C–response/recovery time, C ↓
200–400 nm SnO nanoparticles [37]	N/A	5/5	Resistance	25	S/V ↑ – response time ↓
LaOCI-doped SnO_2_ nanofibers [7]	100–2000	24/92	Resistance	300	Porosity ↑ –response/recovery time↓ Doping ↑ 0–8% –response/recovery time ↓
Carbon nanotubes [58]	500–100,000	A few seconds	Conductance	25	CO_2_ ↑ – pH ↓–I ↑
Poly(ionicliquid) alumina composite [59]	300–3200	420/2400	Impedance	25	CO_2_ ↑ – proton diff ↓

R.H: relative humidity, Diff: diffusion, S.A: surface area, T: temperature, NS: Nanostructure, S/V: surface to volume ratio, I: current, C: capacitance, ↑: increase, ↓: decrease.

**Table 2:** Most significant recent work undertaken on optical nanosensors.

Nanomateiral Used	Sensing Principle	Dynamic Range	Response Time/Recovery Time	Remarks
polydiacetylene nanofibers [110]	Col	400 ppm	instantaneous/NA	Naked eye detection for CO2 using green laser pointers.
Ru nano beads doped HPTS in ormosil matrix. [111]	Col	0–100%	30 s/<60 s	T ↑ – sensitivity ↓ LOD = 0.08%
CuInS2/ZnS quantum dots. [112]	Col	0–100%	23/71 s	T ↑ – sensitivity ↓
Silica nanoparticles porous [113]	Ref	2–5%	48/76 s	porosity ↑–sensitivity ↓
CNTs. [94]	Ref	1000–4000 ppm	3.07/2.95 min	LOD = 75 ppm
Silica nanoflower [114]	Col	400–70,000 ppm	Instananuous/NA	CO_2_ conc. ↑–color intensity ↑
Au-decorated ZnO nanorods [115]	Ref	0–2000 sccm	50/110 s	CO_2_ sensitivity for ZnO–Au lower compared to ZnO
NaYF4:Yb,Er nanoparticles [116]	Col	0–3%	10/180 s	LOD = 0.11%
Cu-benzene-1,3, 5-tricarboxylate [117]	Ref	>500 ppm	40/75 s	Sensing length = 8 cm

Col: colorimetric, Ref: refractometric, ↑: increase, ↓: decrease, LOD: Limit of detection, HPTS: 1-hydroxypyrene-3,6,8-trisulfonate, ormosil: organically modified silica.
